# Treatment of Cardiac Dropsy

**Published:** 1851-02

**Authors:** 


*Treatment of Cardiac Dropsy.*—Dr. Alexander Kilgour, in an interesting paper on Disease of the Heart and the dropsy following, in the *Monthly Journ. Med. Sci.* (Sept. 1850,) makes the following remarks on the treatment of the latter affection:—

"In the treatment of cardiac dropsy, every practitioner has his favorite remedy, taken from the class chiefly of diuretics or purgatives, or both. Of the former, I have found the following combination the most efficient. It pumps the patient out, so to speak, sometimes in a few hours; and it often will do so in repeated attacks of the anasarca.

℞.—Infusi digitalis, ℥iv; acetatis potassæ ʒij; spiritus ætheris nitrosi, ʒij; aquæ cassiæ ℥iss. Capiat cochleare magnum quartâ quâque horâ.

At last there comes an attack in which this and other diuretics cease to act, and we must then fall back on purgatives. Of the latter, unquestionably the most powerful is elaterium. But there surely must be a very great diversity in the strength of this medicine. Some practitioners, from the days of Sydenham, and long before him, downwards, appear to have given it in the dose of two grains, or even more; but I have found a single pill, according to the following formula, generally very powerful:—

℞.—Elaterii. gr. j; extracti colocynthidis comp. ℈ij. ss; extracti hyoscyami, gr. xij. M. Divide in pilulas xij. Capiat unam nocte maneque.

The great objection to the elaterium is the intense sickness, even in this small dose, produced by it. Do the large doses produce less sickness than the smaller? It may be so; but, in the few instances in which I have tried large doses, the sickness was not less. Is there any mode by which this sickening property in this valuable medicine could be removed? The same effect was found by the ancients in the well known and much used, but undeservedly now almost discarded, hellebore; and for the sickness occasioned by it we find it recommended (*Vide* "Oribasius," lib. viii. cap. v.) that, amongst other remedies, the patient should be entertained with a funny little story, or be tossed, like Sancho Panza, in a blanket.* Something more efficient than the former, and less disturbing than the latter, would be a desideratum.

It is the opinion of some that the elaterium acts as a diuretic, as well as a hydragogue chathartic. I remember, when in consultation with Dr. Adams, of Banchory, in a case of cardiac dropsy, having my attention called by him to a formula, where the elaterium was combined with a diuretic, which he had seen prescribed with very great success. I detected it at once as a formula given in Ferriar's valuable "Medical Histories." It acts most powerfully by stool and urine (being composed of several of the most powerful of the diuretics, along with the elaterium); but I always found it to cause much and violent sickness.

℞.—Extracti elaterii, gr. ij; spiritus ætheris nitrosi, ℨij; tincturæ scillæ, oxymeliis colchici, ää ℨss; syrupi rhamni, ℥i. M. Ft. Solutio. Capiat drachmam unam ex aquæ pauxillio, ter, quarterve in die.

The combination of a bitter purgative with a saline one composed of the vegetable alkali and a vegetable acid, is in my experience much more efficient than any single purgative, or than a bitter with a salt formed of a mineral

---

* My learned friend, the translator of Hippocrates and Paulus Ægineta, writes, in a note, in his translation of the latter, "shaken in a garment;" but mine, I assert, is the more spirited version.

acid. The old compound powder of jalap is a well known instance of a mixture of this kind, and is still one of our best purgatives in all dropsies where this class of medicines may be suitable. Ferriar used, as did also Home, a combination of half an ounce of the bitartrate of potass with two grains of gamboge. The infusion of senna with bitartrate of potass is also an old-fashioned and valuable remedy; but the insolubility of the salt is an impediment to the efficiency of this formula. The senna infusion with tartrate of potass, or with the tartrate of potass and soda, is not liable to the same objection; and the advantage of the frequent use of this combination in cardiac disease having a tendency to dropsy, or in the dropsy itself attending that complaint, has been in my hands, and those of my brethren to whom I have recommended it, so unequivocal, that I can speak for it in the highest terms.

The preparations of mercury have proven, no doubt, very successful in the treatment of this form of dropsy, and consequently many practitioners give them a preference. Without wishing at all to detract from the merits of this most valuable agent, I must confess that, in chronic diseases of the heart, I have the same objection to it, and founded on the same grounds, as was that to the celebrated Dr. Fell, or, to use the more classic words of Martial—

> "Non amo te, Sabidi, nec possum dicere quare;
> Hoc tantum possum dicere, non amo te."

I do not like the mercury, and cannot speak from experience of its efficacy in cardiac dropsy.

There comes a time in the treatment of this complaint when not only diuretics in all forms, but even purgatives, cease to remove or even to keep in check the anasarca. And this brings me to speak of another mode of treatment, which often proves palliative for a time—viz., puncturing the lower extremities, and thereby draining off the fluid.

This is not a new mode of treating the disease, though it has at various times fallen into unmerited neglect, and perhaps at the present time more so than at any other. Freind, in his "Historia Medicinæ," refers to the passage in Ætius which treats of this method of curing dropsy. Ætius is quoting from Asclepiades, and says that "an incision is to be made in the internal part of the leg, about four fingers' breadth above the ankle, and that it is to be of the same depth as in venesection. A small quantity of blood flows first, and then there is a continuous discharge of water; and, without inflammation, the wound remains open until the whole dropsy has run off, no internal medicine being used." Freind continues, "Ipsa operatio ab Hippocrate memoratur, et ab ejus temporibus usque ad hunc diem multo sæpe cum successu adhibita est. Aliam puncturæ, scilicet per acum, viam proponit Sylvius de la Boe, hanc a se primo excogitatum glorians; quanquam evidens sit ea omnia ex hac descriptione desumpta esse, atque totidem verbis ab Avicenna expressa." And then he adds, "Verum haud sola hæc inventio recens dicitur, quam antiqui nobis medicinæ scriptores diserte tradiderint. Id autem omnibus in chirurgia vel minimum versatis constat, lanceolam longe præferendam esse cuivis acui, in aperiendis iis, quæ anasarcam comitantur tumoribus."—("Historia Medicinæ," p. 385: Lond., 1733.) Mead commences his notice of the treatment of dropsy ("Monita," p. 130: Lond., 1771) with an account of this operation. He directs an incision to be made two fingers' breadth above the ankle down to the cellular membrane, and no further; and he orders the leg to be fomented with a decoction of emollient herbs, to which some spirits of wine and camphor have been added. He tells us that he has often found this mode of treatment, not only in this disease (anasarca), but also in ascites, of great service, and sometimes curative, the water running out for many days to an extent to exceed all belief. He carefully cautions us to support the patient's strength under a serous discharge from this or any other wound. He then gives a case, apparently hopeless, of anasarca, combined with ascites, where, by a wound made in this manner in each leg, followed by a combination of bitters, squills, and such purgatives as elaterium, calomel and jalap, the patient recovered, and died five years afterwards of another disease.

This method of treatment is also brought under our notice by Heberden, in his "Commentaries," but not with so flattering hopes of advantage. He says, "When these and other diuretics have failed, as is often the case, some have attempted to draw off the water by incisions in the legs, and even by cantharides blisters applied to them; and sometimes blisters take place spontaneously on the legs of dropsical persons. By these means," he continues, "I have seen not a small quantity of water drawn off, but never the disease cured ('morbum autem nunquam sanatum'); but they gave a brief respite, and eased the patient a little." He then notices the great objection to this mode of treatment—the difficulty of healing, and the danger of these wounds—"Odiosum est quod hæc vulnuscula interdum fiunt ulcera sanatu difficilia, vel periculosa, quanquam bis aut ter quotidie pannis laneis ex aqua calida expressis foveantur. Fomenta hæc utilia sunt ad aquam copiosius evocandam."—(Commentarii," p. 191: Londini, 1807.)

I have here quoted the opinions of these very practical men as a test, so far, of the value of the treatment by incisions and puncture. I have no hesitation in saying with both Mead and Heberden, that I have not only often seen it of great benefit to a patient laboring under anasarca, no matter what the pathological cause of that disease, but have also in some cases seen it prove a cure, or, at any rate, that, after recourse had to it, internal medicines, which previously exerted no effect on the dropsical effusion, began to act, and the anasarca did not reappear.

I do not, however, recommend it until all internal remedies have failed. I never have recourse to it till it has become, to use Mead's expression, "unica spes salutis;" and I do not forget the qualification he makes, "et ea dubia." Still I am decidedly of opinion that, whether as a palliative or as a hope of cure, it deserves more notice than now-a-days it seems to me to get.

There are two modes, as stated above, in which the opening for draining off the fluid may be made—either a puncture with the lancet, or with a good stout sewing needle. From a single puncture by the latter, a quantity of water will sometimes run out so as to pass through the bed in a few hours, and require to be collected in vessels placed below it.

Heberden has evidently seen the danger attending this mode of treatment, though he does not say specifically in what it lies. He tells that the wounds are difficult to be healed, but that we need not fear. He also says they are "periculosa." Now, the danger consists in the inflammation of an erysipelatous character, that not unfrequently takes place, extending up the limb, sometimes bringing on *sphacelation*, and for the most part ending in the death of the patient. But if the medical attendant would be satisfied with one incision, and one, or at the most two, punctures, in the proper place, erysipelas is not so apt to occur. The state of the skin—its low vitality during the existence of the dropsy under it—is singularly favorable for this form of inflammation; but a small clean cut, or one or two punctures—and, if there be two, at a distance of not less than two inches from each other—is not so likely to be followed by this as when, in the anxiety to get the water all off speedily, several have been made. The writers whom I have quoted all recommend fomentations to be applied to the wound; but I have often found the evaporating lotion very successful in keeping down any disposition in the part to erysipelas. Frequent ablution, also, with tepid water, and the removal of any cloths that may be soaked with the serum that has run out, will serve to prevent, so far, any irritation from being set up in the skin.

When the operation, even with the needle, has to be often repeated, as is sometimes the case, for the ease of the patient, especially in cardiac dropsy, there is sometimes a degree of inflammatory action set up in the cellular tissue by which its interstices become obliterated, and the skin and it become quite closely adherent to the facia below. I remember this occurring in a gentleman, who had been kept alive for some months by punctures, and who was at last dropsical everywhere, except below the knees, where the adherent and thickened integuments would not distend—thus giving him, however, not a little pain. It has of late been recommended to have rcourse to this treatment much earlier in the disease than was the custom with the old practitioners; and it has been said that the chances of success from it are much greater than when the patient's strength has been exhausted, and the vitality of the skin has been impaired by the long existence of a dropsical effusion. This reasoning is fair; but, whilst admitting it, one practical point may be mentioned, which is, that the fluid does not flow so freely from the incisions as when the areolar tissue has become more open and less resilient by the long existent pressure of the effused serum. And, little as the danger of erysipelatous attacks may be from puncture of the skin in the early stage of anasarca, still there are few that will subject the patient to this chance of danger until all the other more usual and often successful modes of treatment have been tried in vain.

As to another topical means of treatment in this disease —viz., the application of blisters to the anasarcous legs, with the view of draining off the serum—I would have scarcely thought it necessary to speak, believing that almost none, now-a-days, would adopt this practice; but lately I met a very intelligent country practitioner, who told me that he occasionally had recourse to this application. Sydenham has condemned the practice, and branded it as a favorite application of empirics; and he states that "blisters entirely extinguish the natural heat, already almost overpowered by the water and deficiency of the animal spirits, and bring on a gangrene—too common in such cases." Sydenham's authority (and, by the way, he also condemns acupuncture) is great; but I have seen a small vesicle, which had formed on one of the lower limbs, burst and drain off the whole serum from a person affected with extensive anasarca. The case alluded to is one of interest otherwise, though more properly suited to an article on renal dropsy. The patient, a lady, had the most albuminous urine I ever examined. Every remedy for dropsy had been tried by her medical attendant but mercury, and it was only left to me to suggest that, before the patient died, this medicine, which I had never seen more dangerous in this form of dropsy, as has been asserted by writers, than in any other, should be cautiously tried. This was done. Two days afterwards the vesicle above alluded to showed itself and burst, and our patient, much to our surprise, recovered, and is still in good health—seven years after the above attack. What nature does may not always be successful in the hands of art; and the exciting of a vesication by means of a blister is much more likely to be followed by dangerous than by curative effects in all anasarcous limbs, from whatever cause the dropsical effusion may have arisen.—*American Jour. Med. Sci.*

